# Deep Learning Based Evaluation of Spermatozoid Motility for Artificial Insemination

**DOI:** 10.3390/s21010072

**Published:** 2020-12-24

**Authors:** Viktorija Valiuškaitė, Vidas Raudonis, Rytis Maskeliūnas, Robertas Damaševičius, Tomas Krilavičius

**Affiliations:** 1 Department of Control Systems, Kaunas University of Technology, 51423 Kaunas, Lithuania; viktorija.valiuskaite@ktu.edu (V.V.); vidas.raudonis@ktu.lt (V.R.); 2 Department of Multimedia Engineering, Kaunas University of Technology, 51423 Kaunas, Lithuania; rytis.maskeliunas@ktu.lt; 3 Department of Applied Informatics, Vytautas Magnus University, 44404 Kaunas, Lithuania; tomas.krilavicius@vdu.lt; 4 Faculty of Applied Mathematics, Silesian University of Technology, 444-100 Gliwice, Poland

**Keywords:** sperm quality, sperm head detection, convolutional neural network (CNN), deep learning

## Abstract

We propose a deep learning method based on the Region Based Convolutional Neural Networks (R-CNN) architecture for the evaluation of sperm head motility in human semen videos. The neural network performs the segmentation of sperm heads, while the proposed central coordinate tracking algorithm allows us to calculate the movement speed of sperm heads. We have achieved 91.77% (95% CI, 91.11–92.43%) accuracy of sperm head detection on the VISEM (A Multimodal Video Dataset of Human Spermatozoa) sperm sample video dataset. The mean absolute error (MAE) of sperm head vitality prediction was 2.92 (95% CI, 2.46–3.37), while the Pearson correlation between actual and predicted sperm head vitality was 0.969. The results of the experiments presented below will show the applicability of the proposed method to be used in automated artificial insemination workflow.

## 1. Introduction

**Figure 1:**
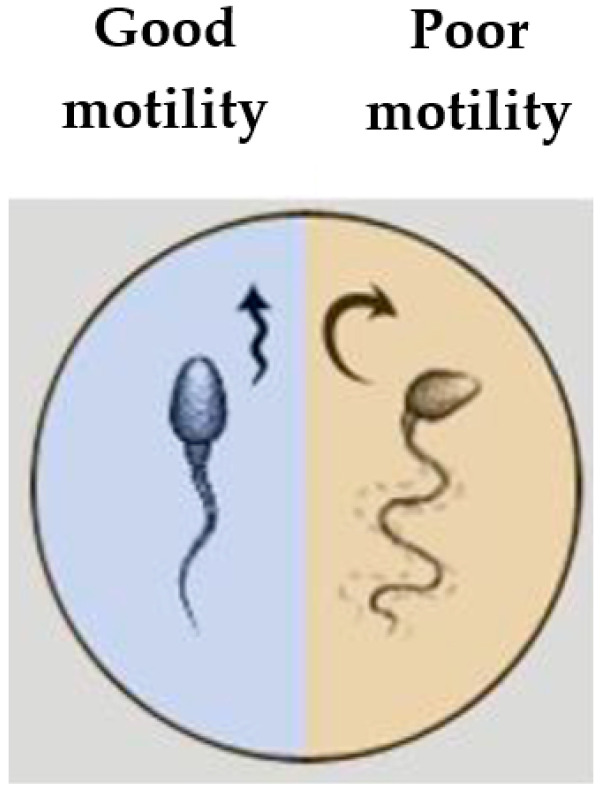
Visual illustration of good and poor motility of a sperm head.

Infertility is recognized clinically when a man and woman are unable to conceive after one year of regular unprotected sex, or when a woman has had two or more failed pregnancies. Around 15–20% of couples face this problem [1]. One of the solutions is artificial insemination. It is a procedure in which a woman′s egg is fertilized by male semen in a test tube rather than in the womb. This method has been used for several decades (the first baby was born after the artificial insemination procedure in 1978 [2]), but the probability of fertilization is still very low; only 30–50% of tests give a positive result. Infertility has previously been considered a woman’s problem, but research has shown that male infertility accounts for up to 47% of cases in infertile couples [3]. Sometimes infertility can be cured by changing one′s habits (e.g., giving up tobacco, alcohol and heavy food) or by removing stress-causing factors. Such changes may favor several crucial quality indicators: sperm motility (the percentage of motile and partially motile sperm should exceed 32% in a healthy sample), volume (normal ejaculation of sperm should be 1.5 mL), viability (a desired value of at least 58%), or concentration (typically about 39 million sperm are found in the sample) [4]. Threshold values for sperm concentration, morphology, and motility can be employed to identify men as subfertile or fertile [5]. Good motility is characterized by the ability of a sperm head to move quickly in one direction, while poor motility is observed when a sperm head moves slowly and in an unorderly fashion (Figure 1). Although infertility can be treated with hormonal medications, such treatment is only effective for a very small proportion of patients, and the treatment itself lasts up to three months.

In cases where neither lifestyle changes nor medication help, artificial insemination is used, i.e., the fusion of a woman′s eggs and a man′s sperm outside the woman′s body, which is implemented using assisted reproductive technology (ART). Possible methods of fertilization are: in vitro fertilization (IVF) [6], where high-quality sperm is poured on the ovum, and intracytoplasmic sperm injection (ICSI), where morphologically healthy-looking sperm is selected with the help of a syringe and injected into the ovum. The latter procedure requires a particularly powerful microscope that would magnify the image up to 8000 times. Not all fertility clinics can afford it, so this method of fertilization is used only in exceptional cases. Although the incidence of intracytoplasmic sperm injection and the percentage of women who give birth are as high as 60%, this procedure is more invasive than IVF, as there is a chance (2%) that the egg will be damaged during sperm injection, making the egg non-viable. In addition, preparing for the ICSI procedure requires more diligence; embryologists, looking through a microscope, must select one healthy sperm and take it with a syringe needle. For this reason, the ICSI method is used only if the IVF procedure does not give the desired results. For artificial insemination, a sample of healthy sperm can be selected with hyaluron binding, centrifugation, magnets, or seemingly simple, compact devices [7]. For the in vitro fertilization procedure, such sampling is sufficient, but the incidence of the procedure itself and the percentage of women who have given birth remain very low, ranging from 30 to 35%. Currently, the selection of sperm cells for artificial insemination takes from 30 min to 120 min [8].

To increase the conception rate of artificial insemination, a computer-aided method to determine the suitability (i.e., motility) of a sperm sample for artificial insemination based on the microscope footage is required. Recently, artificial intelligence (AI)-based methods, such as convolutional neural networks (CNNs) and deep learning combined with computer vision methods, were adopted for multiple biomedical imaging applications, such as disease classification [9,10], edge detection [11], image segmentation [12], knowledge inference [13], image reconstruction [14], shape recognition [15], and others [16].

For ART in general, and for the analysis of sperm quality in particular, the AI techniques have also been adopted for classifying semen data and images [17]. For example, Nissen et al. [18] compared common convolutional neural network (CNN) architectures for human sperm cell-segmentation and recognition in semen sample images, achieving 93.87% precision and 91.89% recall for the best-analyzed network architecture. Hicks et al. [19] predicted the percentage of progressive, non-progressive, and immotile sperm heads from sperm images using ResNet-18 and ResNet-50 models and transfer learning. Movahed et al. [20] used a combined CNN-kmeans-SVM approach to segment the exterior and interior parts of the sperm heads for quantitative morphological analysis of sperm heads. Mohammed et al. [21] adopted a twin support vector machine (TWSVM) for the selection of features from a wider set of texture analysis-based image features, while a back-propagation neural network (BPNN) was used for the classification of healthy/unhealthy human sperm heads, achieving very good results. Their dataset however, was rather small. Thambawita et al. [22] suggested a two-stage architecture, where an autoencoder was used to extract sperm image features, and the pre-trained Resnet-34 CNN was employed for predicting motility and morphology of sperm heads. Butola et al. [23] used feedforward deep neural networks (DNNs) to recognize normal and stress-affected sperm cells. They achieved an accuracy of 85.6% on a dataset of 10,163 interferometric images of sperm cells. Ilhan et al. [24] used multi-stage cascade connected image preprocessing, region-based descriptors and a non-linear kernel support vector machine (SVM) for the recognition of sperm morphology from stained sperm images. The preprocessing techniques such as wavelet-based local adaptive de-noising, overlapping group shrinkage, and automatic directional masking increased the accuracy by up to 10%. In another paper, Ilhan et al. [25] suggested using the Mobile-Net neural network, which has a relatively low number of parameters, for the classification of normal/ab-normal sperms, achieving 87% accuracy. Iqbal et al. [26] developed a custom convolutional neural network (CNN) architecture to categorize human sperm heads, achieving 88% recall on the SCIAN dataset and 95% recall on the HuSHeM dataset. Javadi and Mirroshandel [27] trained a custom CNN to recognize morphological abnormalities of sperm heads and achieved over 83% accuracy in recognizing acrosome, head, and vacuole defects. Kandel et al. [28] used an optimized U-Net network to label the pixels in the sperm image as “head”, “midpiece”, “tail”, or “background”. The results were used to evaluate the dry mass as a proxy for infertility assessment. Riordon et al. [29] adopted VGG16, a deep CNN originally trained on ImageNet, and a transfer learning approach to retrain the network for sperm classification. The study has achieved 94.1% accuracy on the HuSHeM dataset and 62% accuracy on the SCIAN dataset. Shaker et al. [30] adopted a dictionary learning approach to create a dictionary of sperm head shapes. The latter was then employed to categorize the sperm heads into four different classes, achieving 92.2% accuracy on the HuSHeM dataset. McCallum et al. [31] implemented a deep-learning model that employed the VGG1647 network pre-trained on the ImageNet48 dataset with an additional global average pooling layer, succeeded by two fully connected layers. The new model was validated on the dataset of 1064 sperm cell images to predict the DNA Fragmentation Index (DFI).

Similar research has also been performed on non-human sperm, such as cattle or canine semen. Velasco et al. [32] used InceptionResNetV2, Xception, DenseNet121, DenseNet169, MobileNetV1, InceptionV3, and DenseNet201 for the classification of cattle sperm, and they achieved an accuracy of 98.3% on a dataset of 602 images. In another paper, Velasco et al. [33] adopted Inception_ResNetV2 and transfer learning for canine semen evaluation, achieving the highest accuracy of 87%. Hidayatullah et al. [34] adopting a pre-trained DarkNet53 network for sperm-cell detection, achieving 86.91 mAP on the test dataset and a processing speed of 50.3 fps on bull-sperm observation videos. Nevertheless, the use of deep learning neural networks is not fully automatic, in the sense that they still require manual annotation of images used for training a neural network, which is a tedious task in itself [35].

This article proposes a novel deep learning-based algorithm to determine, from a video, whether a semen sample is suitable for an artificial insemination procedure. Our novelty is the application of a Faster R-CNN neural network for sperm head segmentation and identification, combined with a heuristic algorithm for sperm head viability (motility) evaluation.

The remaining parts of the article are as follows: Section 2 describes the dataset used and the methods proposed. Section 3 presents and describes the experimental results. Finally, Section 4 evaluates and discusses the results and presents conclusions.

## 2. Materials and Methods

### 2.1. Dataset

**Figure 2:**
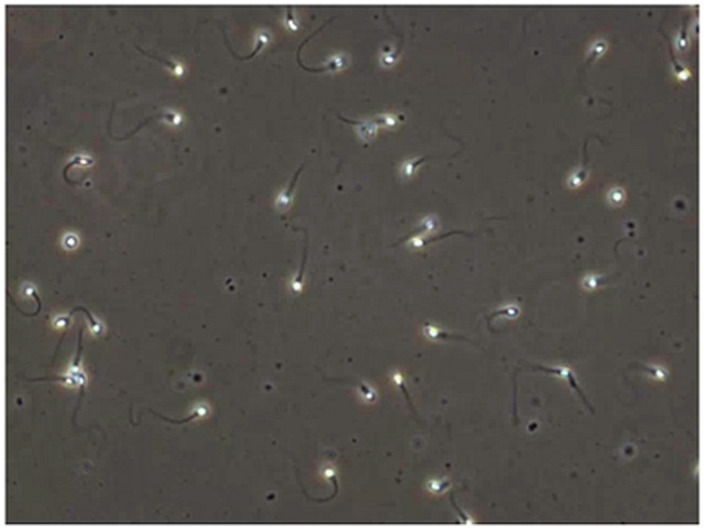
Still image from the sperm sample video.

For our experiments, we used the videos and data variables available from the VISEM-dataset [36]. The Norwegian research laboratory Simula conducted a study that sought to establish a link between a man′s reproductive function and overweight and obesity factors [36]. The embryologists of the study analyzed the sperm samples submitted by the participants and assessed sperm motility, sperm head concentration, total sperm head count, ejaculate volume, sperm morphology, and viability. Samples were examined under a 400-fold magnification microscope with an integrated heated table (37 °C) and captured by a microscope camera (UEye UI-2210C, IDS Imaging Development Systems, Obersulm, Germany). Sample observation videos were recorded for the sperm motility study (see an example of a still image in Figure 2). The dataset contains more than 30 GB of videos, each lasting from two to seven minutes. The videos have a 640 × 480 px resolution and a 50 fps frame rate.

The dataset includes information collected from 85 participants over the age of 18. Sperm analysis was performed for each participant, videos of each sample recorded, sperm fatty acids and their composition were measured, demographic data were recorded, and the results of the spermatogram analysis were performed according to World Health Organization (WHO) requirements, which included single ejaculation volume, sperm color, biological fluid dilution time, and sperm viscosity.

A descriptive table of the main parameters of the dataset to show the median and range of the data is presented in Table 1.

### 2.2. Image Annotation

**Figure 3:**
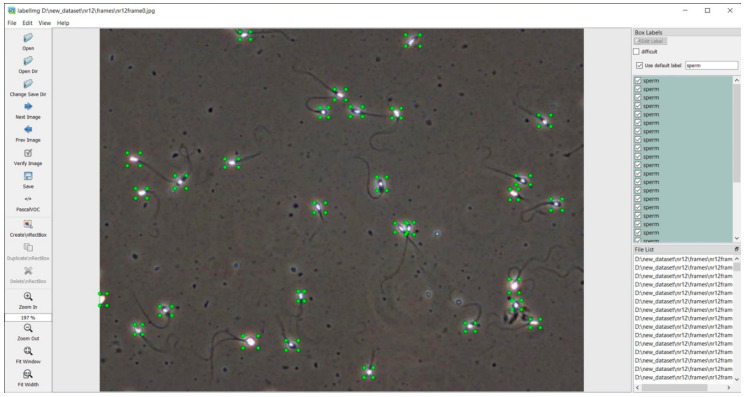
Spermatozoid annotation using LabelImg application.

Image tagging (annotation) is the manual process of recognizing different image elements when tags are added to an image. A separate XML annotation file was created for each image using LabelImg, an image annotation tool. The LabelImg tool uses 2D rectangles to mark an object. An example of the annotation results can be seen in Figure 3. A database of 650 frames with sperm was created to identify the neural network for sperm. An average of 30 sperm heads were recorded in one frame. As only sperm head motility was examined, the sperm tails were not included in the tags, and only the head and neck were marked (Figure 3).

### 2.3. Neural Network

**Figure 4:**
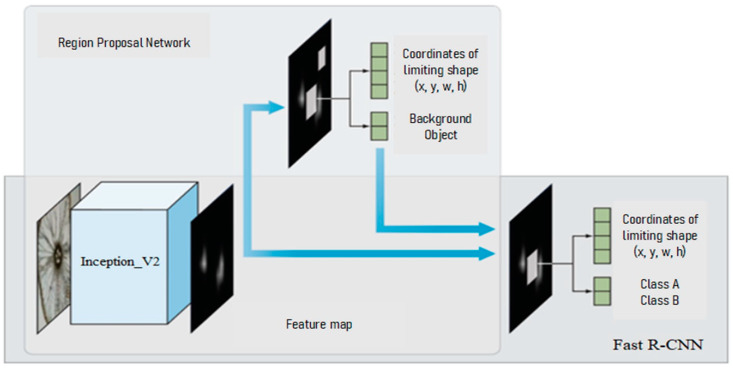
Faster Region Based Convolutional Neural Networks (R-CNN) network architecture.

Faster R-CNN [37], a deep CNN, was applied to each region proposal defined in the previous step, which classified the region proposals into objects and background. The input frame was presented to the network, and a pre-trained convolutional neural network (Inception, Resnet, MobileNet, VGG-16) extracted the characteristics of the input frame. Features were sent to two different components of the Faster R-CNN architecture (see Figure 4).

The Faster R-CNN-Inception_V2-COCO model [37] was used to develop the sperm counting algorithm. Its advantage is that it is quite accurate—able to learn to distinguish objects even from a small database. The model takes about 50 ms to analyze the image. Because the model uses regions, it is easier to detect a larger number of objects in a single frame. Additionally, the R-CNN model can read information from frames of any size, so there is no need to crop frames before submitting them to the neural network (whereas the SSD_MobileNet neural network model only accepts 300 × 300 px frames).

**Figure 5:**
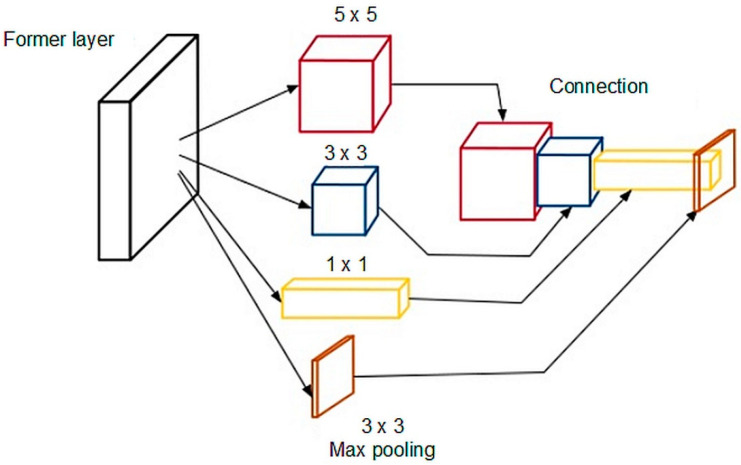
Principle of operation of the Inception model.

The Inception neural network model [38] (see Figure 5) performs several different transformations on the same input frame, combining the results into a single output. For each layer, the 5 × 5, 3 × 3, and 1 × 1 convolutional transformations were performed (features of interest are extracted from the input frame), a 3 × 3 maximum value extraction operator (max pooling) was applied, and the transformation results were output to a single array. The array with the results was passed to another layer, and in the next layer the model decides how and what information to use next.

The parameters of the neural network were set as follows:

- The number of classes is 1 because in the Tensorflow API (Python) the background is not considered as a class, and only objects themselves are counted as a single class;
- The maximum number of first stage proposals—since there can be an average of 30 sperm per frame (the value ranges from several units to 100), the selected value is 100;
- The maximum number of detections per class—the default value is 100. Since there can be on average 30 sperm per frame (value ranges from several units to 100), the selected value is 100;
- The maximum number of total detections—the default value is 100. The project includes one class, depending on the maximum number of detections per class, the selected value is 100;
- Score converter—the sigmoid function is used.

### 2.4. Spermatozoid Tracking Algorithm

**Figure 6:**
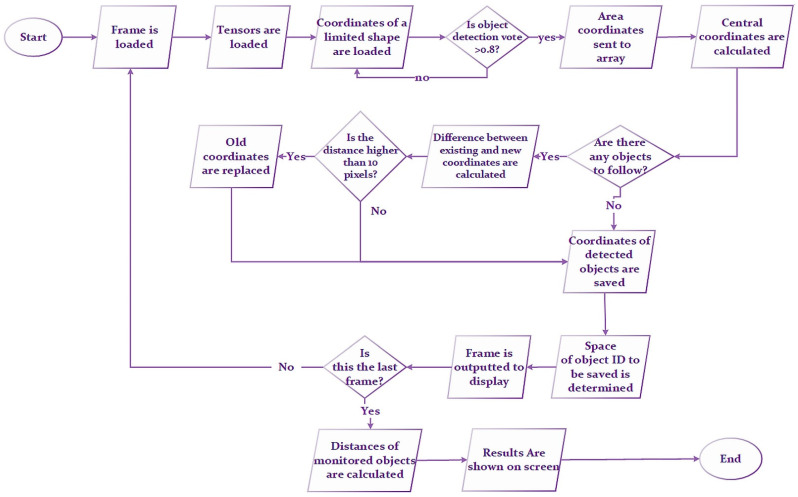
Proposed spermatozoid tracking algorithm.

The proposed active sperm count algorithm is based on tracking the central coordinates of a sperm head. When the program is started, the first frame is scanned (Figure 6). A sperm_track list type variable is created to store the sperm distances traveled. A trained neural network is loaded into the program. The network takes the scanned frame as an input tensor and outputs the output tensor—the coordinates (detection_boxes) of the rectangles bounding the objects (detection_scores), the detected object classes (detection_classes), and the number of detected objects (num_detections).

**Figure 7:**
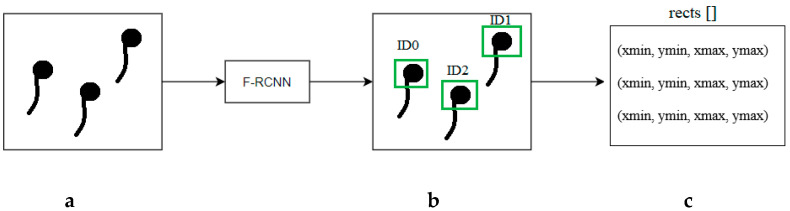
Basic scheme of frame processing: (**a**) sperm sample image, (**b**) segmented images of spermatozoa, (**c**) coordinates of bounding boxes of spermatozoa.

The proposed method (see Figure 7) reads the coordinates of all detected objects (Figure 7b). If the score assigned to an object is greater than the threshold value of 0.8, the coordinates of the bounding boxes are stored (Figure 7c).

**Figure 8:**
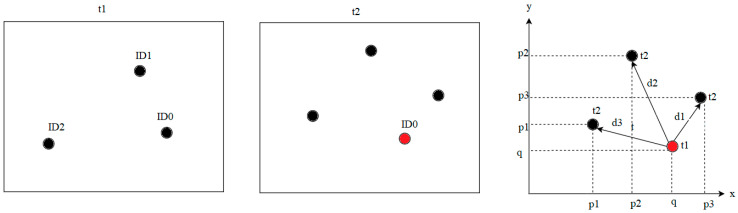
Illustration of the search for the least distant point in the neighborhood of a sperm head.

The center coordinates of the bounding rectangles were calculated (Figure 7c), and the average of the start and end coordinates was derived. The center coordinates were appended to the input_centroids array. Then, the method was performed, checking the dictionary type list of objects; if it was empty, then the program did not track any objects. If the list of objects was not empty, the distance between the coordinates of the input objects and the previously recorded coordinates of objects was calculated (Figure 8) using the Euclidean formula to calculate the distance between two points (Equation (1)):

(1) $$d\left(p,q\right)=\;\sqrt{\overset{n}{\underset{i=1}{\sum }}{\left(q_{i}-\;p_{i}\right)}^{2}};$$

here $q_{i}—$the coordinates of the first point, and $p_{i}—$the coordinates of the second point.

All obtained distances were checked using the following conditions: if the distance was greater than 80 pixels, then the minimum distances were calculated and applied to the corresponding objects according to their ID number. Within one second, a viable sperm must swim at 34.5 µm/s [39]. Since the size of one pixel is 0.0002 m and the videos were filmed under a magnifying microscope of 400 times, which means that the size of one pixel in the database frames is 0.66 µm, the sperm will be considered viable if it traveled a distance of 52 px/s. Sperm vitality is the ratio of the sum of viable sperm to the total distance traveled by sperm, expressed as a percentage. If the activity of the semen sample was greater than 58% [4], the sample was considered to be suitable for insemination. 

## 3. Results

### 3.1. Results of Spermatozoid Viability Evaluation

From the test results of the active sperm counting algorithm shown in Table 2, the viability of only one video clip segment corresponded to that found in the database. The average deviation of the estimated viability of the ten videos from the experimental one was 5.3%. An algorithm that achieves this result is suitable for product development. This may be because only a small portion of the video was being explored. The entire video could not be explored because the location of the video changed several times in each video.

### 3.2. Ablation Study

**Figure 9:**
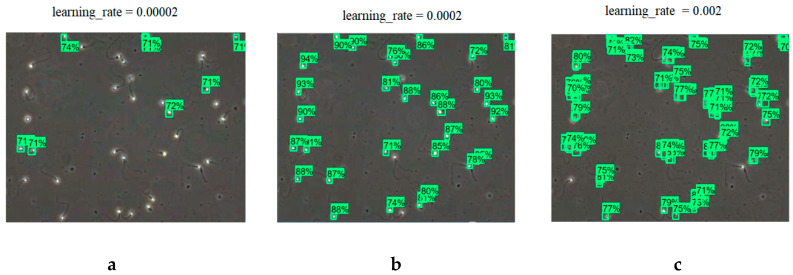
Results of applying different learning speeds: (**a**) learning_rate = 0.00002, (**b**) learning_rate = 0.0002, (**c**) learning_rate = 0.002.

Three learning rates were chosen for training in neural network sperm recognition: 0.00002, 0.0002, and 0.002 (Table 3). In all cases, the neural network learned 2000 steps. The neural network with the lowest learning rate did not have enough steps; it was able to recognize only 20% of the objects in the frame (Figure 9a), while the neural network with the highest learning rate did not absorb the input information, and only 87 objects were found in the same frame (Figure 9c). The neural network with the assigned learning rate of 0.0002 showed the best results; it detected 30 objects in the frame (Figure 9b), the average score offered by the network for the detected object was 0.85. This learning indicator is also applied to further training.

Four iterations were employed to test neural network sperm recognition training (Table 4). After selecting 50,000 iterations, the network detected 30 objects in the frame (Figure 9), the lowest of all calculations being accurate. After selecting 100,000 iterations, the algorithm detected 32 objects out of 33, and the average score for the detected object reached 0.86. After reaching 150,000 iterations, the number of detected objects decreased to 31 out of 33, but the average value of the score for the detected object increased to 0.96. After reaching 200,000 iterations, the algorithm detected all the objects in the frame.

**Figure 10:**
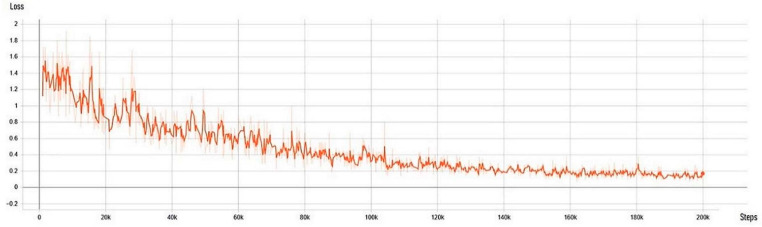
Neural network loss during training.

After 200,000 iterations, the neural network loss dropped to 0.22 (Figure 10). Neural network training lasted 4 days. The loss curve decreased exponentially, which means that the algorithm had successfully learned and adapted to the input task.

The mean accuracy of sperm head detection on the testing dataset was 91.77% (95% CI, 91.11%–92.43%).

**Figure 11:**
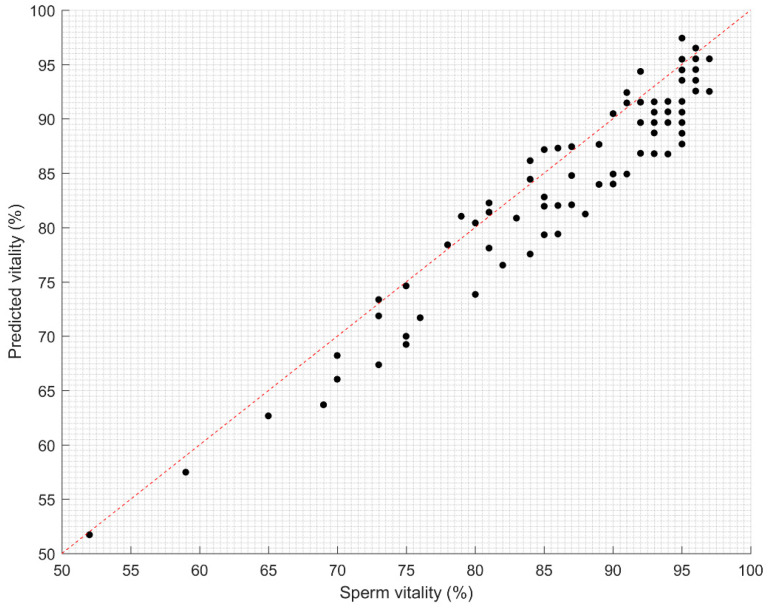
Correlation study of predicted sperm vitality vs. actual sperm vitality. The regression line is plotted (R^2^ = 0.94).

**Figure 12:**
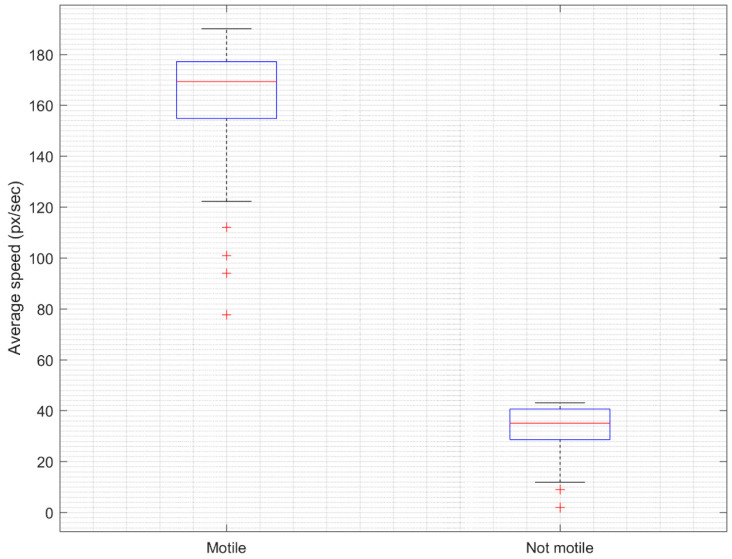
Difference between motile and non-motile sperm heads as determined by the proposed algorithm.

The analysis of the sperm vitality prediction results showed a very good correlation with actual sperm vitality established using laboratory analysis methods (Figure 11). The Pearson correlation coefficient was 0.969 (95% CI, 0.968–0.97). The confidence interval (CI) was established using the bootstrapping method. Furthermore, the statistical significance of the results was tested by a paired t-test, where a p-value of less than 0.05 was deemed to be significant. Usually, the t-test assumes that samples are independent. The difference between motile and non-motile sperm heads was statistically significant (*p* < 0.001), as indicated by the paired t-test. The mean speed of motile sperm heads was 162.5 px/s (95% CI, 157.8–167.2 px/s), while the mean speed of motile sperm heads was 33.35 px/s (95% CI, 31.48–35.22) (see a boxplot representation in Figure 12).

**Figure 13:**
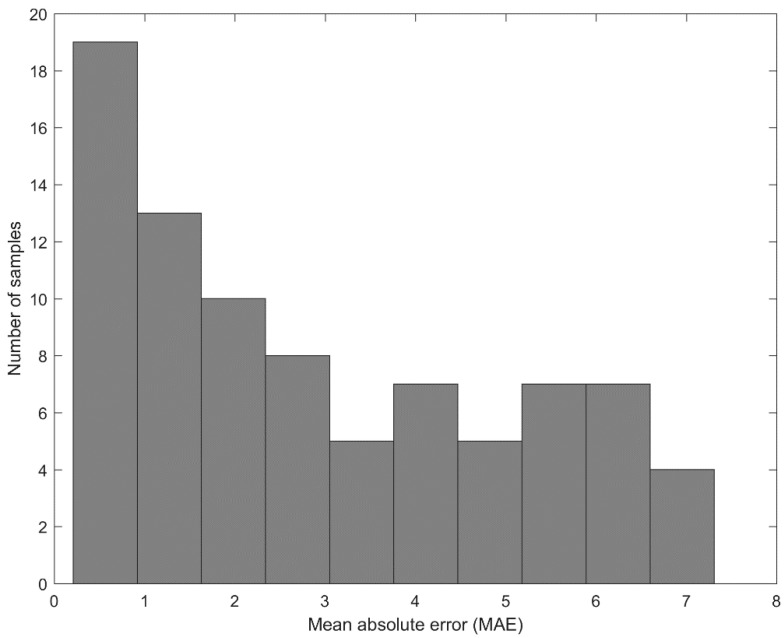
The histogram of mean absolute error (MAE) value distribution.

We also calculated the mean absolute error (MAE) of sperm vitality prediction. Following the suggestion presented in Hicks et al. [19], the results with a mean MAE value below 11 were considered as significant improvements when compared to the ZeroR baseline, which assumes that the predicted values are equal to the average value computed over the dataset. Our results show that in all sperm samples we have achieved an average MAE value of less than 11 (Figure 13), while the grand mean MAE was 2.92 (95% CI, 2.46–3.37). 

## 4. Evaluation and Conclusions

For practical purposes, the classification of human sperm heads can be simplified to a two-class recognition problem, as normal and abnormal sperm heads. In this paper, the criterion for abnormality was the speed of moving sperm heads as an indicator of their motility. The developed deep learning method based on the R-CNN network architecture for sperm head detection was tested on the dataset of sperm videos. The responses of the Faster R-CNN network to different learning rates and number of iterations were investigated experimentally. The algorithm achieved the best results by choosing the learning rate of 0.0002, and the number of iterations of 200,000. These parameters are optimal for the sperm cell image segmentation to efficiently detect small objects such as sperm heads.

Our results show that deep learning neural networks can be used to assess sperm motility consistently and efficiently for the needs of the artificial insemination procedure. The motility of the semen sample calculated by the algorithm differs from the experimental result by only 2.92% on average, while the accuracy of sperm head detection was 91.77%. However, in some cases, our method has failed to correctly recognize sperm heads because of occlusion, similar artifacts, or the sperm head being connected to the video frame border, which made it look like a dark spot. The reason for this result may be that the video fragments used to test the algorithm were too small. The entire video could not be tested because the video area changed several times during the video, and the objects being tracked changed, but the algorithm did not capture the change.

Future work will include the validation of the proposed method on the Human Sperm Head Morphology dataset (HuSHeM) [40].

## Figures and Tables

**Table 1:** Descriptive statistics of the dataset.

Parameter	Statistics	Value
Sperm concentration (×10^6^/mL)	Median (range) Mean ± std	68 (4–350) 82.33 ± 64.11
Average number of sperm heads in one frame	Median (range)	34 (2–175)
	Mean ± std	41.16 ± 32.05

**Table 2:** Testing results of the active sperm counting algorithm.

No.	Frame ID	Objects, Pcs			Experimental Viability, %	Estimated Viability, %	Deviation, %
		Found	Live	Viable			
1	1	80	78	68	85	87	2
2	14	6	5	4	96	84	12
3	22	21	15	10	75	72	3
4	23	10	7	6	85	85	0
5	30	21	20	11	91	87	4
6	42	55	52	43	85	83	2
7	51	90	88	77	86	88	2
8	79	92	86	76	97	88	9
9	81	80	74	64	81	87	6
10	82	39	34	24	85	72	13

**Table 3:** Neural network results of applying different learning speeds.

Learning Speed	Number of Steps	Objects			Average Score
		Mark	Recognize	Wrong	
0.00002	2000	10	7	3	0.71
0.0002	2000	30	30	0	0.85
0.002	2000	87	30	0	0.78

**Table 4:** Results of applying a different number of iterations for neural network training.

Number of Iterations	Detected Objects	Identified Objects	Unrecognized Objects	Accuracy	Average Score
50,000	30	30	3	0.9	0.88
100,000	32	32	1	0.97	0.86
150,000	31	31	2	0.94	0.96
200,000	33	33	0	1	0.97

## Data Availability

Publicly available dataset was analyzed in this study. This data can be found here: https://datasets.simula.no/visem/.
